# The european organization for research and treatment of cancer quality of life questionnaire-BR 23 breast cancer-specific quality of life questionnaire: psychometric properties in a Moroccan sample of breast cancer patients

**DOI:** 10.1186/1756-0500-7-53

**Published:** 2014-01-21

**Authors:** Samira El Fakir, Naima Abda, Karima Bendahhou, Ahmed Zidouh, Maria Bennani, Hassan Errihani, Abdelatif Benider, Rachid Bekkali, Chakib Nejjari

**Affiliations:** 1 Department of epidemiology and public health, Faculty of Medicine, University Sidi Mohammed Ben Abdellah, Fez, Morocco; 2 Fondation Lalla Salma Prevention and Treatment of Cancers, Rabat, Morocco; 3 National Institute of Oncology, Rabat, Morocco; 4 Oncology Center Ibn Rochd, Casablanca, Morocco

**Keywords:** Breast cancer, Quality of life, Reliability, Validity

## Abstract

**Background:**

Quality of life (QOL) and its measurement in cancer patients is becoming increasingly important. Breast cancer diagnosis and treatment are often associated with psychological distress and reduced QoL. In Arabic-speaking countries, QoL of patients with cancer is inadequately studied.

The aim of this study was to test the reliability and validity of the Moroccan Arabic version of the European Organization for Research and Treatment of Cancer (EORTC) Breast Cancer-Specific Quality of Life Questionnaire (QLQ-BR23).

**Methods:**

After translation and cross-cultural adaptation, the questionnaire was tested on breast cancer patients. The participants’ number for the test and the retest were 105 and 37 respectively. Internal consistency was tested using Cronbach’s alpha coefficient (α), the test-retest reliability using intraclass correlation coefficients (ICC). Construct validity was assessed by examining item-convergent and divergent validity.

**Results:**

The questionnaire was administered to 105 patients. The mean age of patients was 48 years (SD: 16), 62.9% were married. 68.6% of all participants lived in urban area.

The average time to complete the QLQ- BR23 was 15 min. Cronbach’s alpha coefficient, were all >0.7, with the exception of breast symptoms and arm symptoms. All items exceeded the 0.4 criterion for convergent validity except item 20 and 23 related to pain and skin problems in the affected breast respectively.

**Conclusion:**

In general, the findings of this study indicated that the Moroccan Arabic version of the EORTC QLQ-BR23 is a reliable and valid supplementary measure of the QOL in breast cancer patients and can be used in clinical trials and studies of outcome research in oncology.

## Background

Worldwide, breast cancer is the most common malignancy diagnosed in women, the second most common type of cancer after lung cancer (10.4% of all cancer incidence, both sexes counted) and the fifth most common cause of cancer death. In 2008, breast cancer caused 458 000 deaths worldwide [1,2]. Breast cancer is the most frequent type of cancer among women in Morocco (33.4%) [3].

Today, Health-related quality of life (HRQOL) is now considered an important endpoint in cancer clinical trials. Although there has been considerable researches on the quality of life (QOL) of women with breast cancer [4], the results may not be significantly affecting clinical decision – making because the clinical significance of findings is not specified [5,6]. More studies are therefore needed to make QOL assessment feasible, understandable and scientifically viable in oncology researches and practices [6]. In particular, HRQOL assessments in oncology facilitate doctor –patient communication, they point to areas where patients may experience serious problems, they can be used as diagnostic tools for problem- oriented follow – up care, and the data are strong predictors of survival [7,8].

The European Organization for Research and Treatment of Cancer (EORTC) has developed several questionnaires to assess QoL. In assessing quality of life in cancer patients, it is recommended to use a cancer-specific questionnaire as a general measure of quality of life (e.g. the EORTC QLQ-C30) [9] plus site-specific modules (e.g. breast cancer specific or gastric cancer specific). Thus, for instance the EORTC, in addition to core cancer quality of life questionnaire, has developed several site-specific questionnaires including one for breast cancer (QLQ-BR23) [10] in order to collect more relevant patient-reported outcomes in studying quality of life in this group of cancer patients. The English-language versions of these questionnaires were successfully tested [10], it has been found to be reliable and valid in diverse cultures, including, the United Arab Emirates [11], Iran [12], China [13], Greece [14], Kuwait [15] and South Asia [16]. The EORTC QLQ-BR23 questionnaire was previously translated into Arabic but was not efficiently validated for use in patients speaking Moroccan dialect. Indeed, the dialect spoken in Morocco is very different from the classical Arabic language which is understood by part of the Moroccan population mainly educated people.

The objective of this study was to translate and adapt the original version of the European Organization for Research and Treatment of Cancer (EORTC) Breast Cancer-Specific Quality of Life Questionnaire (EORTC QLQ-BR23) from the English to the Moroccan Arabic language, to refine its terminology and to adapt it to the Moroccan culture.

### Patients and methods

#### Characteristics of the EORTC QLQ-BR-23original version

The 23 item breast cancer -specific module, the EORTC QLQ-BR-23 [17], consists of two multi-item functional scales (body image and sexual functioning), three symptom scales (systemic side effects, breast symptoms, and arm symptoms), and three single item scales on sexual enjoyment, future perspectives, and upset by hair loss. The responses indicate the extent to which patient has experienced symptoms or problems. Each item was scored on a four point Likert scale (“not at all” [1], to “very much” [4]), and the time frame was “during the past week”, except for the sexual items (“during the past four weeks”).

### Translation and cultural adaptation of the EORTC QLQ-BR 23

The EORTC QLQ-BR 23 was translated into local Moroccan Arabic. We followed the published guidelines for cross-cultural adaptation of health-related quality of life measurement [18,19]. The questions in the original version of the EORTC QLQ-BR 23 were initially translated into Moroccan Arabic by two bilingual people. Then it was reviewed by a committee of professionals composed by oncologists and epidemiologists, in order to obtain the first Arabic version. The resulting version of this intermediate Moroccan Arabic version was applied to volunteers persons with cancer that agreed to participate and to present their opinion if the translated version was comprehensible; two researchers had to register the comments, doubts and suggestions. The second Arabic version was written after this initial assessment. Subsequent back-translation into English was performed by two other translators with a good knowledge of English but who were not familiar with the EORTC QLQ – BR 23. A comparison with the original version was performed by a committee of professionals and translators to check whether they contained literal differences. The translation was then reviewed and adjusted by the committee. In the process of translating the questionnaire from English into Arabic, several items were substituted to adapt the terminology to the local culture : item 39 “Have you felt physically less attractive as a result of your disease or treatment? “ and item 40” Have you been feeling less feminine as a result of your disease or treatment?”. A final version of the EORTC QLQ – BR23 in Moroccan was elaborated. As most of the patients in our sample were illiterate, the questionnaire was administered by two interviewers. Their task was to read out the questions and mark the chosen answers for this category of patients without providing any input.

### The subjects recruitment

Between August and November 2009, subjects were recruited from the two main oncology centers in the country (National institute of oncology in Rabat and oncology center of Ibn Rochd hospital in Casablanca). Subjects attending these two centers are from around the country (north, center and south). Subjects were eligible if they were at least 18 years old, had a confirmed diagnosis of breast cancer and spoke Moroccan Arabic. Ethical approval was obtained from the ethics committees in the University Hospital Center Hassan II in Fez- Morocco and all the subjects were informed of the conditions related to the study and gave their written, informed consent.

### Instruments and procedures

Two questionnaires of the Arabic version of the EORTC QLQ-BR23 were administered to patients by two different interviewers. The order of interviewers was randomly defined.

The participants’ number for the retest were 37. A third questionnaire was administered three to ten days later to assess reproducibility. Participants provided socio-demographic and clinical data and a measure of pain on a visual analogical scale.

### Scoring

The scoring algorithm recommended by the EORTC [20] was used to analyze participants’ responses to the QLQ-BR23 questionnaires. In summary, the algorithm creates an average from component item responses and then transforms this to a value on a 0–100 scale. For the functional scales, a higher score corresponds to better function. For symptom scales, a higher score corresponds to more frequent and/or more intense symptoms.

### Statistical analysis

Descriptive statistics were generated to evaluate missing data, score distributions (i.e. mean, range, floor and ceiling effects). The reliability and validity of the EORTC QLQ-BR23 were examined in the study.

### Reliability

The internal consistency of the Moroccan Arabic version of the EORTC QLQ – BR23 questionnaire was assessed for the multi-item questionnaire scales using Cronbach’s alpha coefficients. A value of 0.70 or greater was considered as adequate [21]. Test-retest reliability and inter-observer reliability were estimated by calculating the intraclass correlation coefficient (ICC) for each of the score components of the EORTC QLQ – BR23.

### Validity

We used multitrait scaling analysis to examine the extent to which the items of the questionnaire could be combined into the hypothesized multi-item scales. This analysis was based on an examination of item-scale correlations for item-convergence and item-discriminance [22]. Evidence of item-convergence was defined as a correlation of 0.40 or greater between an item and its own scale. item-discriminance was satisfied if each item has a substantially higher correlation with its hypothesized scale than with scales measuring other concepts.

### Acceptability

The acceptability of the EORTC QLQ – BR23 was assessed with the response rate, percentage of missing Data and time required to complete the questionnaire. A P value of <0.05 was considered statistically significant. Data analysis was performed using SPSS 17.

## Results

In total, the questionnaire was administered to 105 patients, 44 were recruited in Rabat and 61 in Casablanca. The mean age was 48 (standard deviation 16) years. The majority were married (62.9%); illiterate (60%) and lived in urban areas (68.6%). The Socio-demographic characteristics of the study sample appear in Table 1.

**Table 1:** **Sociodemographic characteristics of 105 patients who completed EORTC QLQ**-**BR23 questionnaire**

	**N**	**%**
**Centre**		
Rabat	44	41.9
Casablanca	61	58.1
**Marital status**		
Single	14	13.3
Married	66	62.9
Widowed	10	9.5
Divorced	15	14.3
**Occupation**		
Employed	6	5.7
Unemployed	3	2.9
House wife	95	90.5
Retired	1	1
**Educational level**		
Illiterate	63	60.0
Primary	22	21.0
Secondary	16	15.2
University	2	1.9
**Residence area**		
Urban	72	68.6
Rural	30	28.6
**Âge** mean (SD)	48.1	(10.5)
**Third assessment (test-retest)**	37	35.2

*SD*: Standard deviation.

The average time to complete the EORTC QLQ-BR23 was 15 min. Missing data rate for items varied from 0.0% to 49.5%. 83% of items didn’t have missing responses.

Data on central tendency and variability of EORTC QLQ-BR23 scale are presented in Table 2. The scores for different scales ranged from 34.0 to 77.8. “Systemic therapy side effects” scale had the lower score (median 57.14). High floor effect was observed for the “Future perspective “scale and the higher ceiling effect was observed for the “Body image” (Table 2).

**Table 2:** **Central tendency**, **variability of the EORTC QLQ**-**BR23 scale**

	**N**	**Median**	**Mean**	**Standard deviation**	**Floor effect**^**a **^**(%)**	**Ceiling effect**^**b **^**(%)**
**Body image**	105	83.33	77.54	25.82	0.0	**39.0**
**Sexual functioning**	66	66.66	61.11	29.83	3.8	13.3
**Sexual enjoyment**	53	66.67	58.49	29.17	5.7	8.6
**Future perspective**	105	66.67	46.98	41.27	**39.0**	24.8
**Systemic therapy side effects**	105	57.14	59.86	22.98	1.0	2.9
**Breast symptoms**	105	83.33	77.86	19.94	0.0	25.7
**Arm symptoms**	105	77.78	0.00	26.64	1.9	22.9
**Upset by hair loss**	50	68.94	34.00	41.23	25.7	9.5

^a^Percentage of the lowest modality.

^b^Percentage of the highest modality.

Data on reliability of EORTC QLQ-BR23 scale are presented in Table 3. Throughout the instrument, only one scale (breast symptoms) did not meet the 0.70 internal consistency criterion, and another (arm symptoms) was borderline (α = 0.68).

**Table 3:** **Internal consistency and reliability of the EORTC QLQ**-**BR23**

	**Cronbach’s α**	**Reliability CCI**	
		**Inter-observer**	**Test-retest**
**Body image**	0,76	0,76	0,85
**Sexual functioning**	0,76	0,5	0,79
**Sexual enjoyment**	-	0,24	0,68
**Future perspective**	-	0,72	0,72
**Systemic therapy side effects**	0,7	0,87	0,68
**Breast symptoms**	0,5	0,80	0,79
**Arm symptoms**	0,63	0,85	0,75
**Upset by hair loss**	-	0,80	0,79

The ICC between two interviewers, ranged from 0.24 for “Sexual enjoyment” to 0.87 for “Systemic therapy side effects “.

We collected test/retest data from 37 patients among whom the questionnaire was administered twice (during the baseline visit and on average 5 days later). Test-retest reliability was assessed using the ICC, which ranged from 0.68 for “Sexual enjoyment” and “Systemic therapy side effects “to 0.85 for “Body image”.

Results for multitrait scaling analysis are shown in Table 4. The breast symptom scale exhibited moderate item-convergence with only two out of 4 items (50.0%) exceeding the criterion of 0.40 for the correlation coefficient (r : 0.04-0.8). The body image and Arm symptoms scales exhibited 100% item-convergence (r: 0.77-0.83 and 0.57-0.84, respectively) and 100% item-discriminance (r : 0.06-0.53 and 0.02-0.52, respectively).

**Table 4:** **Multitrait scaling analyse of the EORTC QLQ**-**BR23**

**Scales**	Convergence (r, % success)*	Discriminance (r, % success)**
**Body image**	0,77-0,83, 100	0.06-0.53, 100
**Sexual functioning**	0,89-0,91, 100	0.09-0.35, 100
**Systemic therapy side effects**	0,48-0,66, 100	0.01-0.37, 96.42
**Breast symptoms**	0,04-0.8, 50	0.00-0.89, 0.75
**Arm symptoms**	0,57-0,84, 100	0.02-0.52, 100

*Number of item-scale correlations greater than 0.40 /total number of item-scale correlations.

**Number of correlations of items with own scales significantly higher than correlations with other scale/total number of correlations.

## Discussion

The EORTC QLQ-BR23 instruments have been translated into local Moroccan Arabic according to procedures documented elsewhere [18,19]. The present study aimed to assess the reliability and validity of this translation for use in Morocco. This evaluation of the measurement properties of the Moroccan Arabic version of the EORTC QLQ-BR23 shows that it is a reliable and valid measure of QoL in Moroccan patients with breast cancer.

In the translation stage, we followed the international guidelines for cross-cultural adaptation of health-related quality of life measures [18,19]. We then supplemented a pilot test in Moroccan patients who were asked to identify words and sentence structures that were problematic for the target population. Next, a conceptual adaptation in the final version was performed: Certain expressions in the original version of the EORTC QLQ-BR23 needed modification in the Moroccan version of the EORTC QLQ-BR23. They mainly concerned the item 39 “Have you felt physically less attractive as a result of your disease or treatment? “ and item 40” Have you been feeling less feminine as a result of your disease or treatment?”. The Arabic dialect translation of the phrases “ less attractive “ and “ less feminine” doesn’t exist, so we have changed the first one into “your look isn’t appreciated anymore”, while we translated the second one into classical Arabic.

The necessary number of subjects used was calculated based on the reliability curve of Streiner [23] and was considered sufficient to evaluate psychometric properties. The mean time required for conducting the interviews was 15 min, the same time reported in studies from other countries [24-26]. Time between test and retest was five days on average. Streiner and Norman indicated that expert opinions regarding the appropriate interval varies from one hour to one year, depending on the task, but generally, a retest interval of 2 to 14 days is usually used [23].

The internal consistency coefficients of most scales in the EORTC QLQ-BR23 were also satisfactory. This was confirmed by the Cronbach’s alpha values that exceeded 0.70 for all scales except the arm symptoms and breast symptoms scales. The highest value of Cronbach’s alpha for the EORTC QLQ-BR23 was found for sexual functioning (alpha: 0.76), a finding previously reported among Iranian and Western women [27]. This suggests that sex-related determinants of QoL are perceived similarly by patients with different cultural backgrounds.

The test/retest correlations of the EORTC QLQ-BR23 were also high in most scale/single items. Because of the small sample size, the results of test/retest of this study, though mostly satisfactory, might be subject to errors and should be read with caution.

Construct validity was assessed by testing convergent and discriminant validity of items. The body imageand sexual functioning scales exhibited strong psychometric properties with satisfactory results for multitrait scaling. The Breast symptoms scale evidenced moderate item-convergence, which is consistent with previous studies on Greek patients with breast cancer [14]. This result for multitrait scaling confirmed the hypothesized scale structure, implying that the translation of the items and the response choices are appropriate and that scale scores derived from the Moroccan Arabic version could contribute to cross-cultural comparisons.

Because of the high frequency of illiteracy among participants, an interviewer had administered the questionnaire for most patients. Unlike northern countries, the questionnaire could not be used as an auto-administered questionnaire except for a minority of Moroccan population. Another limitation related to illiteracy among participants in fact the answers to the questions could be affected especially those related with their sexual life which’s a kind of taboo for them.

A limitation of the study is that responsiveness over time was not performed. We would recommend that additional studies be carried out with patients under active treatment in order to document the responsiveness.

Despite the fact that Arabic language is commonly spoken across the country, there are some other regional languages such as “Tarifit”, “Tamazight” and “Tachelhit” that are more popular in some Moroccan regions. But, the majority of these people speak also Arabic. Further validation should be specifically performed in these regions because inclusion in these patient groups in local or national clinical studies is essential.

## Conclusion

Based on the findings of this study, the Moroccan Arabic version of the QLQ-BR23 questionnaire has acceptable reliability and validity, which are comparable to those reported in other languages. In addition, the questionnaire could be used in clinical trials that evaluate the impact of specific interventions on the QoL of Moroccan patients with breast cancer.

## Abbreviations

EORTC QLQ-Br23: The European Organization for Research and Treatment of Cancer Quality of Life Questionnaires-Breast 23; EQ-5D: Euroquol 5 dimension; ICC: Intraclass correlation coefficient; SF-36: 36-Item short form health survey; VAS: Visual analogical scale; SD: Standard deviation.

## Competing interests

The author’s declare that they have no competing interests.

## Authors’ contributions

SE has contributed to conception and design, acquisition of data, analysis and interpretation of data, have been involved in revising the manuscript critically and have given final approval of the version to be published; NA has contributed to conception and design, interpretation of data and have been involved in drafting the manuscript; KB has made substantial contribution to conception and design of data and has been involved in drafting the manuscript; AZ has been involved in drafting the manuscript and have given final approval of the version to be published. MB has contributed to conception and design of data; HE has contributed to conception and design of data. AB has made substantial contributions to conception and design of data; RB has made substantial contributions to conception and design of data; CN has been involved in drafting the manuscript, revising it critically for important intellectual content and has given final approval of the version to be published. All authors read and approved the manuscript.
